# The Effects of Lumbar Spinal Fusion on Spinopelvic Biomechanics Years After Primary Total Hip Arthroplasty: A Case Report

**DOI:** 10.7759/cureus.100558

**Published:** 2026-01-01

**Authors:** Nicholas K Pappa, Lucas Kasson, Zachary Paragas, Matthew Beal

**Affiliations:** 1 Orthopaedic Surgery, The Ohio State University Wexner Medical Center, Columbus, USA; 2 Orthopaedic Surgery, The Ohio State University College of Medicine, Columbus, USA

**Keywords:** hip-spine syndrome, spino-pelvic parameters, tha complications, tha instability, total hip arthroplasty: tha

## Abstract

Spinopelvic biomechanics are critically important for hip stability following total hip arthroplasty (THA). Lumbar spinal fusion, particularly multilevel fusion, alters the dynamic relationship between the spine, pelvis, and hip, resulting in reduced pelvic mobility and increased risk of prosthetic instability. We present the case of an 85-year-old woman with a well-functioning right THA that remained stable for 11 years until she underwent L2-L5 laminectomy and posterior spinal fusion with L4-L5 transforaminal lumbar interbody fusion. Within the subsequent two years, she experienced two distinct episodes of posterior prosthetic hip dislocation, each occurring during forward flexion. This case highlights the biomechanical consequences of lumbar fusion, reviews the underlying mechanisms of altered spinopelvic mobility, and discusses strategies for preventing and addressing hip instability in patients with spinal stiffness or fusion.

## Introduction

Hip osteoarthritis (OA) is a leading cause of disability and functional decline in older adults, with an estimated prevalence of 8.5% in North America [1]. Total hip arthroplasty (THA) remains the definitive treatment for end-stage OA, reliably improving pain and mobility [2]. However, differentiating symptoms related to hip pathology from lumbar degenerative disease can be challenging. Patients frequently present with overlapping groin, buttock, thigh, and knee pain, an interplay described as hip-spine syndrome [3]. This syndrome underscores the biomechanical interdependence of the spine and hip: pathology in one region can accelerate degenerative changes or alter loading patterns in the other [3].

As the aging population grows, many patients require both THA and lumbar spinal fusion (LSF). LSF is a commonly performed procedure for degenerative spinal conditions and involves the permanent fixation of one or more vertebral segments to eliminate pathologic motion, restore stability, and relieve neural compression. By design, fusion restricts physiologic lumbar flexion and extension, increasing spinal stiffness and limiting the spine’s ability to accommodate postural changes. Up to 40% of individuals undergoing primary THA have coexisting lumbar spondylosis, and approximately 1% of all THA recipients have a stiff or fused lumbar spine at the time of surgery [4,5]. Numerous studies have demonstrated that reduced spinopelvic mobility, particularly after multilevel LSF, is a significant risk factor for postoperative prosthetic instability [6-8].

Normal spinopelvic biomechanics rely on coordinated motion between the lumbar spine, pelvis, and hip to maintain sagittal balance throughout activities such as sitting, standing, and bending [9-11]. When transitioning from standing to sitting, the pelvis naturally retroverts, increasing functional acetabular anteversion and protecting against posterior impingement [12,13]. LSF disrupts this mechanism by restricting lumbar flexion and limiting posterior pelvic tilt, thereby reducing functional anteversion during hip flexion [14].

Although the relationship between LSF and increased THA instability is well documented, the literature contains very few detailed case reports demonstrating late-onset instability in previously stable THAs following spinal fusion. Only one prior case report has described this specific sequence of events [15]. We present a similar case of recurrent posterior instability arising 11 years after an uncomplicated THA, occurring only after multilevel lumbar fusion. This case provides an opportunity to examine the biomechanical consequences of spinal rigidity and emphasizes the importance of spinopelvic assessment in the management and prevention of THA instability.

## Case presentation

An 85-year-old woman with a history of hypertension, hyperlipidemia, and type 2 diabetes mellitus underwent a right THA in 2014 for symptomatic end-stage OA unresponsive to conservative therapy. The procedure was performed via a posterolateral approach. The gluteus maximus was bluntly split, the short external rotators were tagged and released, and a capsulotomy was performed to facilitate dislocation.

Implants included a cemented Zimmer CPT femoral stem (size zero) with a 28-mm +0 femoral head and a 46-mm acetabular component containing a 10° lipped polyethylene liner. A dome screw was placed for supplemental fixation. Intraoperative trialing demonstrated a stable construct through a full arc of motion. The arthrotomy and soft tissues were repaired in a layered fashion. Her recovery was uncomplicated, and she remained asymptomatic without instability for 11 years postoperatively.

In 2023, she underwent L2-L5 laminectomy and posterior spinal fusion with L4-L5 transforaminal lumbar interbody fusion (TLIF) for lumbar stenosis with neurogenic claudication and degenerative spondylolisthesis at L3-4 and L4-5.

**Figure 1:**
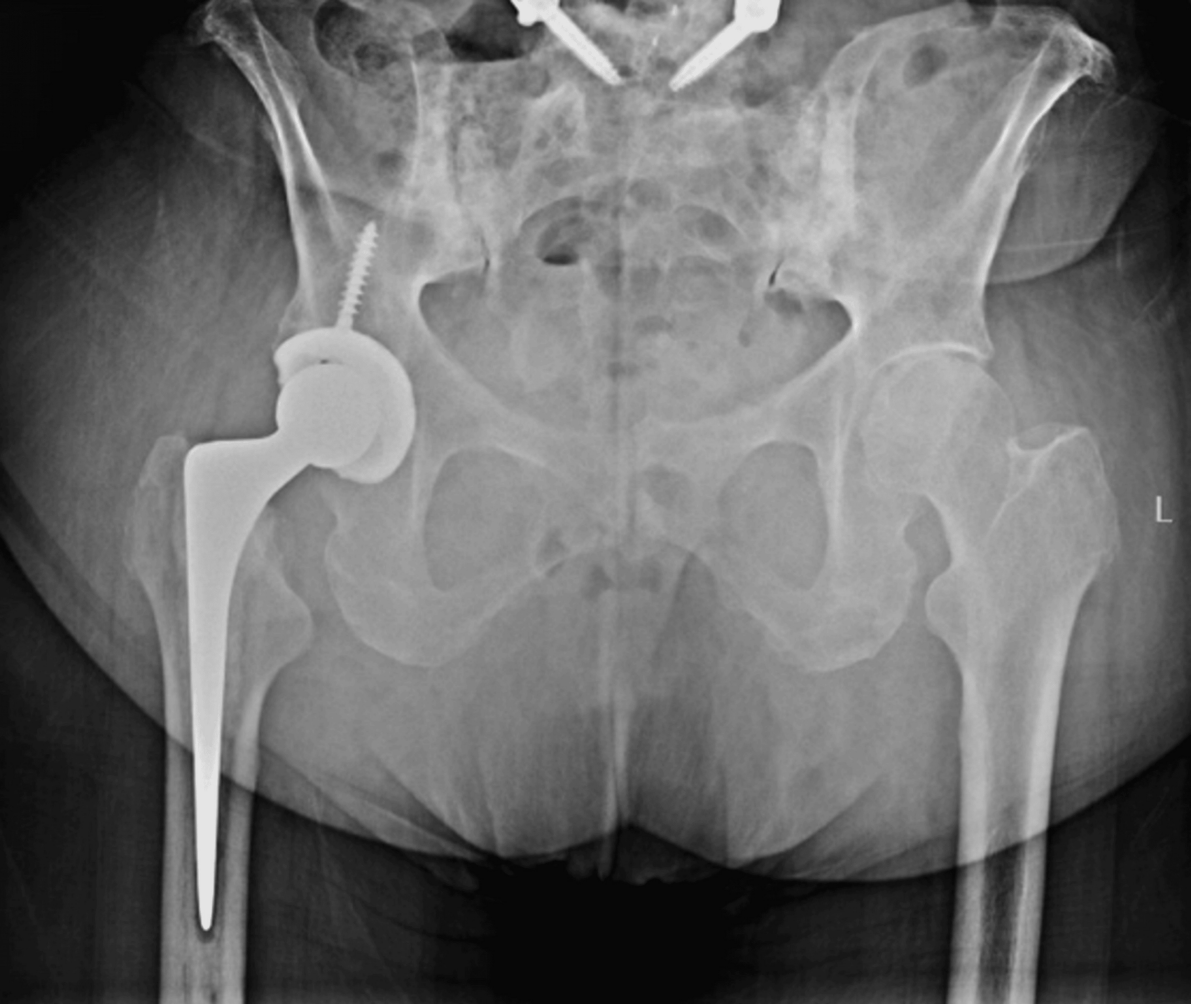
Anteroposterior pelvis radiograph of the right total hip arthroplasty demonstrating intact hardware with no signs of loosening, status post closed reduction following a first-time dislocation.

Two years later, in early 2025, the patient sustained her first posterior prosthetic dislocation while bending forward to retrieve an object. Radiographs following closed reduction confirmed concentric relocation and no evidence of component loosening (Figure 1). She was managed with an abduction brace and instructed on posterior hip precautions.

**Figure 2:**
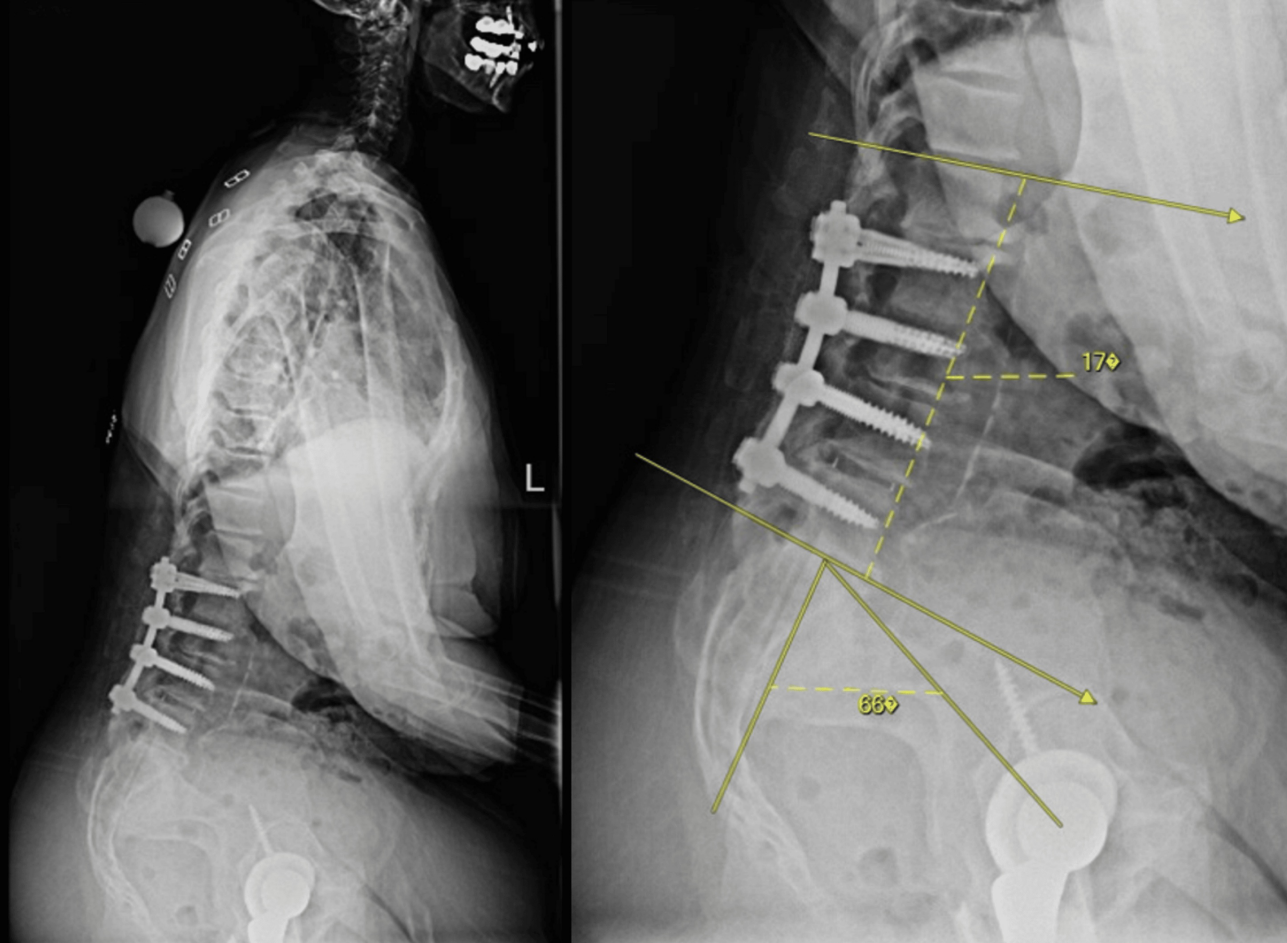
Spinopelvic parameter measurements are shown on this patient’s radiograph. A lumbopelvic mismatch is highlighted, with a pelvic incidence of 66° and lumbar lordosis of 17°, resulting in a difference of 49°, which is far greater than 10° and places the patient at a significantly higher risk of posterior dislocation.

Several months later, she experienced a second posterior dislocation, again occurring during trunk flexion while leaning forward. Closed reduction was again successful. Given her history of multilevel fusion and suspected alterations in functional acetabular anteversion, revision surgery was recommended because it appeared that after the patient’s L2-L5 fusion, her radiographic pelvic incidence-lumbar lordosis (PI-LL) mismatch was 49°, as seen in Figure 2, far exceeding the 10° threshold associated with increased instability risk; this represented profound sagittal imbalance and markedly diminished compensatory pelvic motion (Figure 2). However, the patient declined operative management and opted for continued conservative treatment with bracing and activity modification.

## Discussion

The coordinated interaction between the lumbar spine, pelvis, and hip forms a tightly integrated biomechanical chain essential for maintaining stability following THA [6]. Under normal conditions, transitioning from standing to sitting requires approximately 20-30° of posterior pelvic tilt accompanied by lumbar flexion, collectively increasing functional acetabular anteversion and preventing prosthetic impingement [13,14]. This dynamic spinopelvic adaptation is critical for safely accommodating hip flexion throughout daily activities.

Lumbar degenerative disease and, more definitively, LSF disrupt this adaptive mechanism by limiting lumbar flexion and attenuating posterior pelvic tilt. As a result, patients experience a functional loss of acetabular anteversion in flexed positions, predisposing them to posterior impingement and instability, particularly during activities involving trunk flexion [5,15]. The severity of this effect is strongly correlated with the number and location of fused segments. Multilevel fusions involving the lumbosacral junction (L4-S1) produce the greatest reduction in pelvic mobility, generating a rigid spinopelvic segment that is unable to compensate during sitting or bending [16,17].

Importantly, the patient’s THA remained stable for 11 years prior to her spinal fusion, underscoring that implant position alone did not predispose her to instability. Instead, the fusion created a new biomechanical environment in which a previously well-functioning acetabular orientation became functionally "retroverted" during flexion. This phenomenon, described as dynamic malpositioning, is well recognized among patients with stiff or fused spines and has been reinforced by multiple large registry and meta-analysis studies demonstrating a three- to 10-fold increased dislocation risk in patients with prior lumbar fusion [7-9].

Hip-spine syndrome adds further complexity, as degenerative lumbar pathology may alter spinopelvic mechanics even before surgical intervention. Patients with preexisting lumbar stiffness demonstrate reduced pelvic accommodation and are at greater risk of instability following either THA or fusion [18]. Notably, Pirkle et al. reported that lumbar fusion independently increased the likelihood of requiring future THA, reinforcing the reciprocal biomechanical relationship between rigid spines and progressive hip degeneration [18]. This bidirectional interaction underscores the significance of evaluating sagittal balance and spinopelvic motion as part of preoperative THA planning.

The timing of procedures also influences outcomes. Bala et al. found that patients undergoing LSF prior to THA exhibited significantly higher rates of dislocation than those who underwent THA first [15]. Their work supports the increasingly accepted paradigm that spinopelvic motion, rather than static imaging alone, should guide acetabular component positioning. Standing and sitting lateral radiographs allow surgeons to quantify functional pelvic tilt and identify patients who require modified acetabular anteversion targets or enhanced stability constructs [13,15].

In cases of recurrent instability, such as the present case, revision THA should be tailored to the altered spinopelvic mechanics. Proposed strategies include reorientation of the acetabular cup based on functional imaging, adoption of dual-mobility articulations, or use of constrained liners in select patients [12,19]. Recent biomechanical studies also suggest that dual-mobility constructs may offer great protective benefit among patients with prior lumbar fusion [19]. In rare cases where fixed sagittal imbalance drives posterior instability, corrective spinal realignment has restored stability, though this approach remains controversial and is typically reserved for profound deformity [20].

Our patient declined revision surgery, but her case illustrates the delayed yet profound consequences of altered spinopelvic mechanics following multilevel fusion. The two dislocation events, both occurring during trunk flexion, occurred only after the spine was rendered rigid, supporting the mechanism of functional loss of anteversion rather than traditional positional or soft-tissue etiologies.

Ultimately, this case reinforces that THA stability is not determined by hip factors alone but is intimately tied to spinal alignment and mobility. Given the growing prevalence of patients with both hip and spine pathology, preoperative and postoperative management of THA must incorporate dynamic spinopelvic evaluation to identify high-risk patients, guide implant positioning, and tailor revision strategies when instability occurs.

## Conclusions

LSF can substantially alter spinopelvic biomechanics, reducing compensatory pelvic motion and increasing the risk of posterior prosthetic hip dislocation, even many years after a previously stable THA. Dynamic spinopelvic assessment is essential when evaluating instability in patients with prior fusion. Understanding these biomechanical interactions can guide prevention strategies, optimize implant positioning, and inform the management of recurrent instability.
